# Association between sociodemographic factors and cholesterol-lowering medication use in U.S. adults post-myocardial infarction

**DOI:** 10.1371/journal.pone.0281607

**Published:** 2023-02-09

**Authors:** Elizabeth T. Mansi, Samantha Banks, Alyson J. Littman, Noel S. Weiss

**Affiliations:** 1 School of Public Health, University of Washington, Seattle, Washington, United States of America; 2 Usher Institute for Population Health Sciences and Informatics, University of Edinburgh, Edinburgh, United Kingdom; 3 Seattle Epidemiologic Research and Information Center, Department of Veterans Affairs Puget Sound Health Care System, Seattle, Washington, United States of America; 4 Seattle-Denver Center of Innovation for Veteran-Centered and Value-Driven Care, Health Services, Seattle, Washington, United States of America; Wingate University, UNITED STATES

## Abstract

**Introduction:**

Cholesterol-lowering medications offer effective secondary prevention after myocardial infarction (MI). Our objective was to evaluate the association between sociodemographic factors and cholesterol-lowering medication use in high-risk adults.

**Methods:**

We conducted an analysis using weighted data from 31,408 participants in the 2017 and 2019 Behavioral Risk Factor Surveillance Systems cross-sectional surveys, who had a self-reported history of MI and high blood cholesterol. The sociodemographic factors evaluated were sex, age, race and ethnicity, annual household income, education level, relationship status, and reported healthcare coverage. We estimated the weighted prevalence of medication use, and weighted prevalence differences (with 95% confidence intervals) across categories, adjusting for sex, age group, healthcare coverage, smoking status, hypertension, and diabetes.

**Results and discussion:**

Overall, 83% of survey participants with a self-reported history of both MI and high blood cholesterol reported currently using a cholesterol-lowering medication. The prevalence of use was only 61% in those without self-reported healthcare coverage, compared to 85% of those with healthcare coverage (adjusted prevalence difference of -20%; 95% CI: -25% to -14%). Use of cholesterol-lowering medication was relatively low in younger adults and higher in older adults, leveling off after age 65 years. The proportion of Native Hawaiian or Pacific Islanders who were using a cholesterol-lowering medication was relatively low, but otherwise there was little variation by race and ethnicity. Household income, education level, and relationship status were weakly or not associated with medication use.

**Conclusions:**

Knowledge of characteristics of persons who are relatively less likely to be adherent with cholesterol-lowering medications for secondary prevention may be useful to policymakers and healthcare providers involved in the long-term treatment of MI patients. Policy makers might consider a reduced cost prescription coverage for persons without current healthcare coverage who have sustained an MI to reduce future cardiovascular morbidity and mortality.

## Introduction

About 805,000 Americans experience a myocardial infarction (MI), also known as a heart attack, every year [1]. There are an estimated 8.8 million Americans with a history of MI (3.1% prevalence based on 2015–2018 data) [2] who are at a particularly high risk of hospitalization and death due to subsequent ischemic events, including MI or stroke. Clinical practice guidelines published by the American Heart Association and the American College of Cardiology (AHA/ACC) recommend cholesterol-lowering medications (e.g. statins) in all MI survivors for secondary prevention [3]. Cholesterol-lowering medications lower low-density lipoprotein-cholesterol (LDL-C) by reducing cholesterol synthesis (e.g., statins) or decreasing cholesterol absorption (e.g., bile acid sequestrants) [4]. High blood cholesterol is considered a preventable risk factor for atherosclerosis and MI [5, 6]. Non-adherence to clinical guideline-recommended medications after MI leads to avoidable morbidity and an excess burden to the healthcare system. Previous studies on statin adherence post-MI have limitations, including small study size [7–9] or relatively short follow-up period post-MI (e.g., 1–12 months) [10–15]. Most studies have used prescription as proxy for actual use, and were restricted to populations older than 65 years and/or limited to a specified healthcare coverage (e.g., Medicare patients or a single source of administrative claims data) [10, 12–14, 16–18]. Additionally, many of the previous studies lacked information on sociodemographic factors such as income, education level, or relationship status. The presence of information on associations between sociodemographic characteristics and medication underuse in MI survivors could potentially help guide clinicians to focus on those individuals with the highest likelihood for medication underuse.

The present study used data collected by the Behavioral Risk Factor Surveillance System (BRFSS) [19], which included a large sample of adults representative of the United States population. We studied associations between demographic characteristics, healthcare coverage, and current use of cholesterol-lowering medication among people with a self-reported history of MI and high blood cholesterol.

## Methods

The BRFSS is a national cross-sectional survey conducted in all 50 states, Washington DC, and three US territories with over 400,000 respondents annually. The BRFSS is a state-based system designed to collect information via telephone interview in English or Spanish on health conditions, health behaviors, healthcare access, and other factors. The BRFSS sample was drawn from both landline and cellphone numbers, and used a modified random digit dialing system to disproportionately include numbers that are thought to be households (landlines). A weighting system was employed to account for the complex sampling and non-response. Our study utilized data from the core 2017 and 2019 surveys that was asked of all respondents, as those were the most recent datasets available with ascertainment of cholesterol-lowering medication use at the time of our study. Research using BRFSS data is exempt from IRB review because it is not considered human subjects research, is publicly available, and contains no identifiable information.

**Fig 1:**
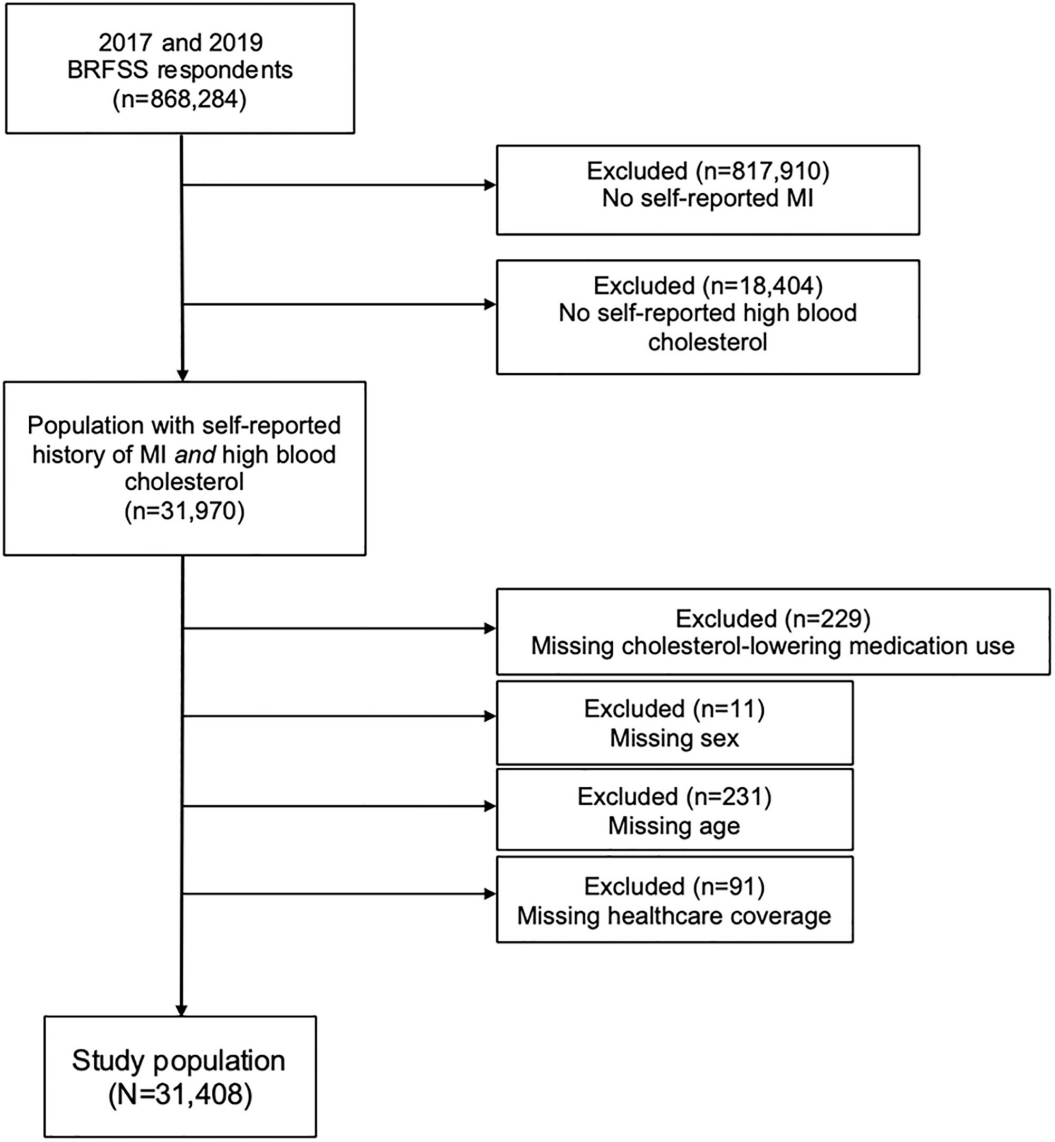
Participant flow diagram. BRFSS: Behavioral Risk Factor Surveillance System; MI: myocardial infarction.

Our study population consisted of adults 18 years and older with a self-reported history of both MI *and* high blood cholesterol level (method for determining history described below) because those without self-reported high blood cholesterol were not asked about cholesterol-lowering medication use. Of the 868,284 respondents who completed the 2017 and 2019 surveys, 50,374 participants had a self-reported history of MI. We excluded 18,966 people *without* a self-reported history of high blood cholesterol (and/or missing data on history of cholesterol-lowering medication use, sex, age, or healthcare coverage), bringing our final analysis population to 31,408 (Fig 1), reflecting over 6 million adults living in the United States.

Our main variables of interest were demographic and socioeconomic characteristics, including sex, age, race and ethnicity, annual household income, education level, and relationship status. Age of the respondent was classified into one of the following groups: 18–34 years; 35–44 years; 45–54 years; 55–64 years, 65–74 years; and 75+ years. We created a combined race and ethnicity variable by first categorizing respondents as Hispanic or non-Hispanic. Non-Hispanic survey respondents were classified by their race as American Indian/Alaska Native, Asian, Black, Native Hawaiian/Pacific Islander, White, multiracial, and other race. Income was defined as self-reported annual household income from all sources. We collapsed annual household income into four categories: 1) less than $25,000; 2) $25,000 up to $50,000; 3) $50,000 up to $75,000; and 4) $75,000 or higher. Highest education level completed was divided into the following four groups: 1) did not graduate from high school; 2) high school graduate; 3) some college or technical school; and 4) college or technical school graduate. For relationship status, we grouped those self-reported as never married, separated, divorced, and widowed into one category (“single”), and grouped married or member of an unmarried couple into another group (“in a couple”) based on the survey options. We assessed the presence of healthcare coverage from the survey question, “Do you have any kind of healthcare coverage, including health insurance, prepaid plans such as HMOs, or government plans such as Medicare, or Indian Health Service?” This was categorized as either “yes” or “no.”

We considered smoking status, history of hypertension, and history of diabetes as potential confounding variables. Each of these is a risk factor for MI and can be associated with sociodemographic characteristics. Smoking status was categorized as: 1) never; 2) former; or 3) current smoker. History of hypertension was based on the survey question, “Have you ever been told by a doctor, nurse or other health professional that you have high blood pressure?” A history of diabetes was based on the survey question, “Have you ever been told by a doctor, nurse or other health professional that you have diabetes?” They were both categorized into one of three groups: 1) no; 2) borderline or only during pregnancy; or 3) yes.

Our outcome of interest was current cholesterol-lowering medication use. This information was obtained from the survey question “Are you currently taking medicine prescribed by your doctor or other health professional for your blood cholesterol?” This question was only asked of participants who previously answered “yes” to the question, “Have you ever been told by a doctor, nurse or other health professional that your blood cholesterol is high?”

Prior to analysis, we excluded all participants with missing covariate data of interest (Fig 1). Descriptive statistics of sociodemographic variables from all study population respondents were generated unweighted, as well as weighted percentages to account for the complex survey design of BRFSS. We fit log-binomial generalized linear models to estimate the weighted prevalence differences (PD) of cholesterol-lowering medication use. Potential confounders were assessed. Sex, age group (defined here as either under 65 years or 65 and older), healthcare coverage, smoking status, history of hypertension, and history of diabetes were identified as confounders and adjusted for in the final regression models used to calculate the adjusted PD. Statistical analyses were performed with R v4.0.3 using the *survey* package.

## Results

Population characteristics of the 31,408 survey participants, representing over 6 million U.S. adults, with a self-reported history of MI and high blood cholesterol are shown in Table 1. Sixty-three percent were men, and 72% of all participants identified as non-Hispanic White. The overall weighted prevalence of cholesterol-lowering medication use was 83.2% (95% CI: 81.7% to 84.8%), with 80.5% of women and 84.9% of men reporting current use (adjusted PD = -4.9%; 95% CI: -6.7% to -3.0%). Except for persons under 45 years of age and those without healthcare coverage, a substantial majority reported current use of such medication, irrespective of other measured sociodemographic characteristics. We found a lower prevalence of medication use in persons under 65 years of age than in older persons, with use steadily being more common during the decades leading to age 65 (Table 2). Other than the relatively small sample of participants identifying as Native Hawaiian or Pacific Islander, in whom the prevalence of cholesterol-lowering medication use was 61%, differences according to race and ethnicity were small. Black survey participants had a 3% higher prevalence of medication use compared to non-Hispanic Whites (adjusted PD = 3.3%; 95% CI: 0.6% to 6.0%). In contrast, Hispanic survey participants had a 5% lower prevalence of medication use compared to non-Hispanic Whites (adjusted PD = -5.2%; 95% CI: -9.3% to -1.1%). For persons with a reported annual household income less than $50k, the prevalence of cholesterol-lowering medication use was modestly lower than those reporting income above $50k. Lastly, the prevalence of medication use was 20% lower among those who did not report having any healthcare coverage compared to those who did (PD = -20.2%, 95% CI: -25.7% to -14.8%).

**Table 1:** Population characteristics among 2017 and 2019 BRFSS participants (unweighted and weighted) with self-reported history of myocardial infarction and high blood cholesterol.

	Unweighted Totals N = 31,408 (%)	Weighted Totals N = 6,642,133 (%)
**Sex**		
Female	12,693 (40)	2,481,504 (37)
Male	18,715 (60)	4,160,629 (63)
**Age (years)**		
18–34	173 (0.6)	100,449 (1.5)
35–44	625 (2.0)	276,102 (4.2)
45–54	2605 (8.3)	871,143 (13)
55–64	7309 (23)	1,807,339 (27)
65–74	10838 (35)	1,966,022 (30)
75+	9858 (31)	1,621,077 (24)
**Race and Ethnicity**		
American Indian or Alaska Native (NH)	811 (2.6)	119,946 (1.8)
Asian (NH)	257 (0.8)	168,369 (2.6)
Black (NH)	2272 (7.2)	671,634 (10)
Hispanic	1615 (5.1)	740,370 (11)
Native Hawaiian or Pacific Islander (NH)	117 (0.4)	12,408 (0.2)
Multicultural (NH)	705 (2.2)	95,186 (1.5)
Other (NH)	247 (0.8)	40,268 (0.6)
White (NH)	24721 (79)	4,640,963 (72)
Missing	663	
**Annual Household Income (in USD)**		
< 25,000	10862 (35)	2,448,107 (44)
25,000–50,000	7204 (23)	1,382,214 (25)
50,000–75,000	3368 (11)	664,381 (12)
75,000+	4642 (15)	1,026,157 (19)
Missing	5332	
**Education Level**		
< High School	4043 (13)	1,523,071 (23)
High School Graduate	10230 (33)	2,048,010 (31)
Some College or Technical School	9095 (29)	1,977,087 (30)
College or Technical School Graduate	7957 (25)	1,080,435 (16)
Missing	83	
**Relationship Status** ^a^		
Single	15,838 (50)	3,082,771 (47)
In a Couple	15,436 (49)	3,537,141 (53)
Missing	134	
**Healthcare Coverage** ^b^		
No	1421 (4.5)	442,169 (6.7)
Yes	29987 (96)	6,199,963 (93)
**Smoking Status**		
Never	10,586 (35)	2,121,089 (33)
Former	13,768 (45)	2,766,656 (44)
Current	5927 (20)	1,470,564 (23)
Missing	1127	
**History of Hypertension**		
No	5265 (17)	1,122,232 (17)
Borderline, or only during pregnancy	242 (1.0)	62,963 (1.0)
Yes	25,817 (82)	5,424,882 (82)
Missing	84	
**History of Diabetes**		
No	18,036 (58)	3,692,903 (56)
Borderline, or only during pregnancy	1114 (3.6)	291,817 (4.4)
Yes	12,196 (39)	2,639,140 (40)
Missing	62	

Those missing information on sex, age, and healthcare coverage were excluded from the study population.

^a^ Relationship Status: “Single” includes never married, separated, divorced, and widowed; “In a Couple” includes married or member of an unmarried couple.

^b^ Healthcare Coverage: Respondents who answered yes to having any kind of healthcare coverage, including health insurance, prepaid plans such as HMOs, or government plans such as Medicare, or Indian Health Service.

*NH*: Non-Hispanic, *USD*: United States Dollar.

**Table 2:** Weighted prevalence, unadjusted, and adjusted prevalence differences (PD) of current cholesterol-lowering medication use among BRFSS participants with self-reported history of myocardial infarction and high blood cholesterol.

	Weighted Prevalence of Medication Use % (95% CI)	Unadjusted PD % (95% CI)	Adjusted PD % (95% CI)
**Sex** ^a^			
Female	81 (79 to 82)	**-4.4 (-6.3 to -2.5)**	**-4.5 (-6.4 to -2.7)**
Male	85 (84 to 86)	Reference	Reference
**Age (years)** ^b^			
18–34	34 (20 to 48)	**-56 (-70 to -42)**	**-46 (-58 to -33)**
35–44	61 (53 to 68)	**-28 (-36 to -21)**	**-24 (-32 to -16)**
45–54	74 (71 to 78)	**-15 (-19 to -11)**	**-11 (-15 to -7.6)**
55–64	83 (81 to 85)	**-6.3 (-8.4 to -4.3)**	**-4.3 (-6.4 to -2.3)**
65–74	89 (88 to 90)	Reference	Reference
75+	88 (87 to 89)	-1.0 (-2.6 to 0.7)	-1.7 (-1.9 to 1.6)
**Race and Ethnicity** ^c^			
American Indian or Alaska Native (NH)	77 (71 to 82)	**-8.3 (-14 to -2.4)**	**-7.1 (-13 to -1.1)**
Asian (NH)	83 (73 to 93)	-1.7 (-12 to 8.0)	1.8 (-10 to 10)
Black (NH)	85 (83 to 88)	0.2 (-2.4 to 2.8)	2.0 (-0.9 to 4.8)
Hispanic	75 (71 to 79)	**-9.6 (-14 to -5.4)**	**-5.1 (-9.2 to -1.0)**
Native Hawaiian or Pacific Islander (NH)	61 (36 to 85)	-24 (-49 to 0.6)	-21 (-46 to 4.2)
Multicultural (NH)	79 (73 to 86)	-5.5 (-12 to 1.1)	-4.4 (-11 to 2.5)
Other (NH)	84 (76 to 92)	-0.9 (-8.5 to 6.8)	1.2 (-6.9 to 9.3)
White (NH)	85 (84 to 86)	Reference	Reference
**Annual Household Income (in USD)** ^c^			
< 25,000	82 (80 to 83)	**-5.2 (-7.9 to -2.5)**	**-4.5 (-7.3 to -1.8)**
25,000–50,000	83 (81 to 85)	**-3.8 (-6.8 to -0.90)**	**-5.9 (-8.8 to -2.9)**
50,000–75,000	87 (84 to 89)	0.1 (-3.1 to 3.3)	-1.3 (-4.5 to 1.8)
75,000+	87 (85 to 89)	Reference	Reference
**Education Level** ^c^			
< High School	80 (78 to 83)	**-4.8 (-7.9 to -1.7)**	-1.9 (-5.9 to 1.2)
High School Graduate	85 (83 to 86)	-0.3 (-2.6 to 2.1)	1.3 (-1.1 to 3.6)
Some College or Technical School	83 (81 to 84)	-2.3 (-4.7 to 0.1)	-1.3 (-3.8 to 1.2)
College or Technical School Graduate	85 (83 to 87)	Reference	Reference
**Relationship Status** ^c^			
Single	81 (80 to 82)	**-4.4 (-6.2 to -2.6)**	**-3.2 (-5.0 to -1.3)**
In a Couple	85 (84 to 87)	Reference	Reference
**Healthcare Coverage** ^d^			
No	61 (55 to 66)	**-24 (-30 to -19)**	**-20 (-25 to -14)**
Yes	85 (84 to 86)	Reference	Reference

^a^Adjusted for age (over/under 65 years), healthcare coverage, smoking status, history of hypertension, and history of diabetes.

^b^Adjusted for sex, healthcare coverage, smoking status, history of hypertension, and history of diabetes.

^c^Adjusted for sex, age (over/under 65 years), healthcare coverage, smoking status, history of hypertension, and history of diabetes.

^d^Adjusted for sex, age (over/under 65 years), smoking status, history of hypertension, and history of diabetes. Results in **bold** are those with p-values <0.05.

*CI*: Confidence Interval, *NH*: Non-Hispanic, *USD*: United States Dollar.

## Discussion

In a large, representative sample of American adults with a self-reported history of MI and high level of blood cholesterol, we found overall high levels of cholesterol-lowering medication use. Certain demographic factors and the lack of healthcare coverage were associated with a considerably lower prevalence of use. Particularly, we found that persons under age 65 years (and especially those under 45 years) had a relatively low prevalence of cholesterol-lowering medication use post-MI. Women also had a somewhat lower prevalence of use when compared to men, consistent with the results of several previous studies [12–14, 17, 20, 21]. The finding that Hispanic respondents had a lower prevalence of cholesterol-lowering medication use when compared to non-Hispanic White respondents is also consistent with previous publications [10, 14, 17]. However, in contrast to previous work [10, 11, 14, 16, 17, 20], we found that Black respondents did not have a lower prevalence of cholesterol-lowering medication use post-MI when compared to White respondents. Other sociodemographic variables that were examined in our study were weakly or not associated with cholesterol-lowering medication use post-MI. Lastly, we found no association between education level and current cholesterol-lowering medication use in adults post-MI.

Publications on cholesterol-lowering medication use in adults under 65 years are limited. In 2010, a systematic review published by Mann et al. [22] found that adults under the age of 50 years had the lowest statin adherence of the age groups examined. However, this study was not limited to persons having survived an MI. Only two previous U.S. population-based studies evaluated medication use post-MI which included adults without healthcare coverage [11, 21]. Mathews et al. [11] conducted a large interview-based study of 7955 MI survivors across over 200 U.S. hospitals assessing several factors in association with medication adherence 6 months post-MI. They found that those with private insurance had a lower risk of non-adherence in the 5 prescribed medication classes post-MI (including statins) when compared to uninsured participants (OR 0.86; 95% CI 0.76 to 0.95). Shah et al. [21] used the 1999–2012 National Health and Nutrition Examination Surveys (NHANES) and reported increasing prevalence of cholesterol-lowering medications in that time frame in adults with a self-reported history of MI, with the prevalence of use over 80% in 2011–2012. While NHANES does include the uninsured [23], the authors did not examine this as a covariate.

Our study has several strengths. We included a large U.S. population-based sample, allowing us to estimate associations across a wide age-range of participants (18–80+ years) with little missing data. Because we used a population-based sample, we were able to estimate the prevalence of current use among people that lacked healthcare coverage and/or were under the age of 65 years. The nature of the BRFSS cross-sectional survey allowed us to estimate prevalence and associations without being restricted to a fixed time period following MI (a common limitation of administrative database studies). This is useful because treatment is recommended indefinitely post-MI. Since our outcome of interest was ascertained through an interview question about current medication use, recall bias is less of a concern. Additionally, as our study used information based on interview and included a large sample size, we were able to have a more detailed assessment of race and ethnicity (<1% classified as “other”) and included annual household income, education level and relationship status—factors not commonly available in previous publications. Lastly, unlike most other studies, our study included all medications (not just statins) understood by the study participant to be cholesterol-lowering.

Our study also has limitations. The question pertaining to our outcome of interest was only asked of survey participants who answered “yes” when asked if they had ever been told they had high blood cholesterol. This restricted our study population to only those with self-reported high blood cholesterol, even though cholesterol-lowering medications are recommended for all people with a history of MI, regardless of cholesterol levels. Second, because all the study variables were self-reported, some degree of misclassification is possible. We cannot confirm the history of MI or confirm that the medication taken was indeed a cholesterol-lowering medication. Finally, because the study was restricted to patients with a history of an MI who remained alive at the time of the survey, survivor bias may be present.

## Conclusions

The data suggest that during 2017–2019, over 80% of Americans who had sustained an MI and reported elevated blood cholesterol levels were using a cholesterol-lowering medication, in agreement with clinical guidelines. Non-use was relatively more common in younger persons and those without healthcare coverage. Knowledge of those persons with a history of an MI and an elevated blood cholesterol level who are relatively less likely to be adherent with cholesterol-lowering medication use may be useful to healthcare providers involved in the long-term treatment of MI patients. Policy makers might consider a reduced cost prescription coverage for persons without current healthcare coverage who have sustained an MI.
